# Self-declared stock ownership and association with positive trial outcome in randomized controlled trials with binary outcomes published in general medical journals: a cross-sectional study

**DOI:** 10.1186/s13063-017-2108-z

**Published:** 2017-07-26

**Authors:** Alberto Falk Delgado, Anna Falk Delgado

**Affiliations:** 1 Department of Surgical Sciences, Uppsala University, Uppsala, Sweden; 2 Department of Clinical Neuroscience, Karolinska Institute, Stockholm, Sweden; 3 Ing. 78/79, Plastikmottagningen, Uppsala University Hospital, Akademiska sjukhuset, 75185 Uppsala, Sweden

**Keywords:** Randomized controlled trials, RCTs, Industry funding, Conflict of interest, Employment, Stock ownership

## Abstract

**Background:**

Describe the prevalence and types of conflicts of interest (COI) in published randomized controlled trials (RCTs) in general medical journals with a binary primary outcome and assess the association between conflicts of interest and favorable outcome.

**Methods:**

Parallel-group RCTs with a binary primary outcome published in three general medical journals during 2013–2015 were identified. COI type, funding source, and outcome were extracted. Binomial logistic regression model was performed to assess association between COI and funding source with outcome.

**Results:**

A total of 509 consecutive parallel-group RCTs were included in the study. COI was reported in 74% in mixed funded RCTs and in 99% in for-profit funded RCTs. Stock ownership was reported in none of the non-profit RCTs, in 7% of mixed funded RCTs, and in 50% of for-profit funded RCTs. Mixed-funded RCTs had employees from the funding company in 11% and for-profit RCTs in 76%. Multivariable logistic regression revealed that stock ownership in the funding company among any of the authors was associated with a favorable outcome (odds ratio = 3.53; 95% confidence interval = 1.59–7.86; *p* < 0.01).

**Conclusion:**

COI in for-profit funded RCTs is extensive, because the factors related to COI are not fully independent, a multivariable analysis should be cautiously interpreted. However, after multivariable adjustment only stock ownership from the funding company among authors is associated with a favorable outcome.

**Electronic supplementary material:**

The online version of this article (doi:10.1186/s13063-017-2108-z) contains supplementary material, which is available to authorized users.

## Background

Randomized controlled trials (RCTs) financed by industry (for-profit) are associated with more favorable outcomes compared with other types of funding sources [1], partly explained by the usage of surrogate endpoints (e.g. biochemical, imaging) among trials financed by industry [2]. Furthermore, declared conflict of interest (COI) among authors of clinical trials has been associated with favorable study conclusions [3–5]. To increase transparency in medical research, the International Committee of Medical Journal Editors (ICMJE) developed, in 2009, an electronic form of financial disclosure to establish uniform reporting of competing interests with the submission of manuscripts, today mandatory in most journals. The form includes, among others, the reporting of personal fees, employment, royalties, stock ownership and grants in order for the reader to consider each potential relationship between author and industry [6].

Most of the previous work evaluating COI and its effect on outcome was performed before the introduction of structured disclosure forms, which are now mandatory in most journals [7]. More recently, it was shown that studies on interventional cardiology devices with industry employees among authors were more likely to report favorable outcomes [8]. The impact of COI on RCT study outcome is, however, still unclear. Further, it has not been established how different type of COIs might impact outcome in RCTs.

We performed a cross-sectional study to evaluate the prevalence and types of conflicts of interest in published randomized controlled trials (RCTs) in general medical journals with a binary primary outcome and to assess the association between conflicts of interest and favorable outcome.

## Methods

An experienced librarian conducted a systematic search identifying all RCTs published in three general medical journals: *Journal of the American Medical Association*, *The Lancet*, and the *New England Journal of Medicine* from January 2013 to September 2015, as previously reported [9]. The search was conducted by using the following expanded search terms: *JAMA*, *Lancet*, *N Engl J Med*, and randomized OR randomized and filtering for 2013/01/01 and 2015/09/23. One author (AlFD) reviewed the titles and abstracts of all potential studies for inclusion. The following inclusion criteria were used: RCTs, parallel-group studies with a binary outcome. We considered a study to be randomized if the term random was mentioned in the methods section of the published article. The study was conducted according to the PRISMA checklist (Additional file 1). The following exclusion criteria were used: letters, abstracts, editorials, non-RCTs, and multi-arm-RCTs. Studies with a continuous variable as primary endpoint were excluded. Financial relationship was defined according to the definition reported in the ICMJE COI form, with relevant COI directly related to for-profit organizations and linked to the submitted work [10]. A study was reported as having COI if any of the authors had a disclosure of COI. Data extraction was performed by one author (AlFD). Data extraction was performed for type of COI, funding source, and study outcome in a blinded manner. Study outcome was assessed first without knowledge of funding and COI. Then COI and funding were extracted blinded for outcome. Studies were classified as having a positive/negative study outcome based on being statistically significant in favor of the intervention compared with the control group in relation to the study primary outcome. All data were double-checked by one author (AnFD). COI was extracted from published papers or online supplements. The following COI types are included in the electronic ICMJE disclosure form if applicable: grants, personal fees, financial support, other, pending patent, issued patent, licensed patent, royalties, stock ownership, and employee. We only considered self-declared COI in the study. This study was not registered in Prospero since the outcome was not related to patient or clinical outcome [11].

## Statistical analysis

We compared all trial variables between the different funding sources (non-profit, mixed profit, and for-profit); non-profit was defined as reference, using odds ratio (OR) and Fisher’s exact test. Pre-specified analyses of factors (COI and funding source) potentially influencing the study outcome were evaluated with binomial simple and multivariable logistic regression. In the regression model, the dependent variable was outcome (positive/negative) and the independent variables were: grants (yes/no), personal fee (yes/no), financial support (yes/no), other (yes/no), pending patent (yes/no), issued patent (yes/no), licensed patent (yes/no), royalties (yes/no), stockholder (yes/no), employee (yes/no), and funding source (for-profit/non-profit/mixed). Variables with a *p* value less than 0.2 in the univariable model were kept in the multivariable model. Unadjusted and adjusted ORs were reported with 95% confidence intervals (CIs). Interactions in the model were explored. Interaction terms with dummy variables were used to test for significant interactions between funding source and all COI subtypes in the logistic regression model. The goodness of fit in the logistic regression model was evaluated with Hosmer-Lemeshow test. All *p* values were two-tailed, with significance defined as *p* < 0.05. All statistical tests were performed using SPSS software (version 20, SPSS Inc., Chicago, IL, USA).

## Results

### Description of included studies

**Fig. 1:**
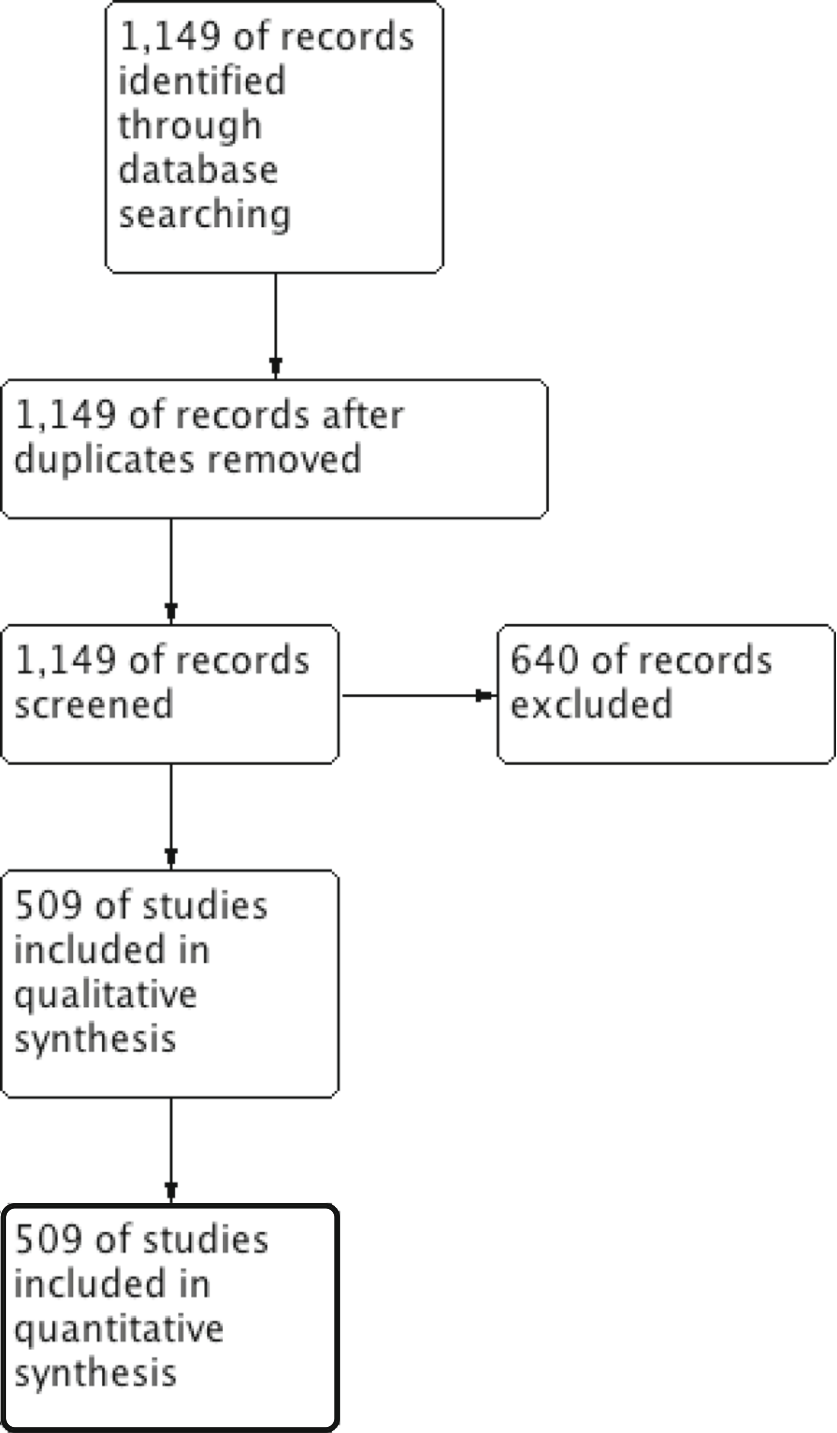
PRISMA flow chart

The electronic search retrieved 1149 hits. A total of 509 consecutive parallel-group RCTs met the inclusion criteria, as previously reported (Fig. 1) [9]. There were no missing data regarding COI or funding, 48% of the studies were funded by non-profit, 23% were mixed funded, and 29% were funded by for-profit organizations. A full description of the prevalence of COI, COI subtypes and funding source are presented in Table 1 and Additional file 2. COI related to any of the authors was reported in 50% of the studies. Out of these, 55% had a favorable study outcome.

**Table 1:** Funding source and conflict of interest

	Non-profit	Mixed funding		For-profit	
	n = 244	n = 117		n = 148	
	n (%)	n (%)	*p* value	n (%)	*p* value
COI present (any)	20 (8)	86 (74)	<0.01	147 (99)	<0.01
Type of COI					
Grant	3 (1)	62 (53)	<0.01	117 (79)	<0.01
Personal fee	8 (3)	62 (53)	<0.01	130 (88)	<0.01
Financial support	5 (2)	30 (26)	<0.01	75 (51)	<0.01
Other	0	13 (11)	<0.01	62 (42)	<0.01
Pending patent	4 (2)	6 (5)	0.08	6 (4)	0.15
Issued patent	1 (0)	4 (3)	0.06	10 (7)	<0.01
Licensed patent	4 (2)	7 (6)	0.04	8 (5)	0.05
Royalties	2 (1)	2 (2)	0.46	4 (3)	0.17
Stock ownership	0 (0)	7 (6)	0.02	74 (50)	<0.01
Employee	0 (0)	13 (11)	<0.01	113 (76)	<0.01

Studies stratified based on funding (non-profit, mixed funding, or for-profit) and COI. Comparison of differences in OR between funding sources, with non-profit studies set as reference.

*COI* conflict of interest

The prevalence of COI varied depending on funding. Any type of COI was found in 99% of for-profit funded RCTs (*p* < 0.01 compared with non-profit), 74% of mixed funded RCTs (*p* < 0.01, compared with non-profit), and in 8% of non-profit RCTs. Personal fees were reported in 88% of for-profit RCTs (*p* < 0.01 compared with non-profit), 53% in mixed (*p* < 0.01 compared with non-profit), and 3% of non-profit RCTs. Stock ownership was reported in in 50% of for-profit funded RCTs (*p* < 0.01 compared with non-profit), in 7% of mixed funded RCTs (*p =* 0.02 compared with non-profit), and none of the non-profit RCTs. For-profit RCTs had employees from the funding company as authors in 76% (*p* < 0.01 compared with non-profit), and mixed-funded RCTs in 11% (*p* < 0.01 compared with non-profit).

### Univariable logistic regression and association with favorable outcome

Table 2 shows the association between a favorable outcome and a specific type of COI with the related effect size. Univariable logistic regression revealed that a favorable outcome was more common with the presence of the following COI: grant, personal fee, other, stock ownership, employee, mixed/for-profit funding.

**Table 2:** Association of industry financial COI and outcome (dependent variable), after univariable binomial logistic regression

COI (any author, n studies)	Favorable outcomes, n (%)	Unadjusted OR for a favorable outcome (95% CI)	*p* value
Grants (182)	104 (57)	2.05 (1.42–2.96)	<0.01
Personal fee (200)	110 (55)	1.85 (1.29–2.65)	<0.01
Financial support (110)	59 854)	1.50 (0.98–2.29)	0.06
Other (99)	66 (67)	2.91 (1.83–4.62)	<0.01
Pending patent (16)	10 (63)	2.02 (0.72–5.64)	0.18
Issued patent (15)	10 (67)	2.43 (0.82–7.22)	0.11
Licensed patent (19)	11 (58)	1.66 (0.66–4.20)	0.28
Royalties (8)	3 (38)	0.71 (0.17–2.99)	0.64
Stock ownership (81)	61 (75)	4.54 (2.64–7.80)	<0.01
Employee (126)	82 (65)	2.86 (1.88–4.36)	<0.01
Funding source			
Non-profit (244)	93 (38)	Reference	
Mixed (117)	47 (40)	2.51 (1.53–4.14)	<0.01
For-profit (148)	93 (63)	2.74 (1.80–4.19)	<0.01

*COI* conflict of interest

### Multivariable logistic regression and association with favorable outcome

The effect of COI after multivariable logistic regression analysis revealed that only stock ownership was more likely associated with a favorable outcome (OR = 3.53, 95% CI = 1.59–7.86, *p* < 0.01) compared with studies without stock ownership in the funding company. Hosmer-Lemeshow test revealed *p* = 0.46 for goodness-of-fit for the model, indicating a good fit of the model (Table 3). In the multivariable logistic regression model, there were no associations between a favorable outcome and any of the other types of COI or funding source. No significant interaction between any of the factors in the logistic regression model were found.

**Table 3:** Association of industry financial conflict of interest with outcome (dependent variable), after multivariable adjustments (binomial logistic regression)

COI (any author)	Adjusted OR for a favorable outcome (95% CI)	*p* value
Grants	1.35 (0.74–2.45)	0.32
Personal fee	0.89 (0.48–1.64)	0.70
Financial support	0.63 (0.36–1.11)	0.11
Other	1.51 (0.74–3.09)	0.26
Pending patent	1.94 (0.52–7.30)	0.33
Issued patent	0.78 (0.19–3.25)	0.78
Stockholder	3.53 (1.59–7.86)	<0.01
Employee	0.79 (0.32–1.96)	0.61
Funding source		0.37
Non-profit	Reference	
Mixed	0.63 (0.27–1.45)	0.27
For-profit	1.02 (0.57–1.82)	0.95

*COI* conflict of interest

## Discussion

Authors of industry-sponsored parallel-group RCTs published in 2013–2015 in three general medical journals report extensive COIs, in particular personal fees received from the funding source, employment in the funding company, and stock ownership in the funding company. Multivariable analysis showed that stock ownership among authors were associated with a favorable study outcome. This is to our knowledge the first study to describe the structure and composition of COI where we report that stock ownership and positive outcomes are associated after multivariable adjustment.

All included papers reported on conflict of interest in the published manuscript or this information could be retrieved from the corresponding online supplementary material. This is a substantial improvement compared with approximately 0.5–2% COI reported almost 20 years ago [12, 13]. Given this historic under-reporting of COI, the structure and composition of financial disclosure has been mainly unknown. In our material, 99% of profit-funded research reported COI. Previous studies based on publications in the top-tier journals in 2000–2008 reported that COI was present in 75% of industry-sponsored studies [7], the contemporary higher prevalence can be associated with more accurate reporting of financial disclosures.

Few publications have focused on associations between different COI domains and favorable outcome. Employment of the funding company was recently shown to be associated with favorable outcome in studies on cardiology interventional devices [8]. Employment was associated with favorable outcome in the univariable analysis in our study, but not after adjusting for the other factors in the model. This difference might be explained by our inclusion of different COI subtypes and adjustment for this in the model. Stock ownership among authors has previously not been linked to more favorable outcome [7], possibly explained by a previous under-reporting of disclosures [14]. In univariable analysis several of the COI domains (grants, personal fee, financial support, other, stockholder, employee, mixed funding, and for-profit funding) were significantly more likely associated with a positive outcome. After adjusting for COI subtype and funding source, there was only one significant remaining effect (stock ownership). Stock ownership among any of the authors were associated with a favorable outcome. Our results are in contrast with previous studies that have shown that for-profit are associated with favorable outcomes [3, 4]. Compared with previous studies [3, 4, 7], we included COI subtypes and funding sources in a multivariable model allowing for adjustments for possible interactions between variables. Stock ownership was almost exclusively reported in for-profit sponsored studies. However, no significant interaction was found in the model after exploring between funding source and COI domains.

Stock ownership is a special type of COI since a positive outcome of a trial might directly result in an increase in the company’s stock price compared with, for example, a personal fee for conducting a study that does not necessarily result in future revenues depending on the outcome of the study. Stocks for employees are commonly used to attract and keep the employee within the company and function as economic incentive to profit from any market price rise. Half of all industry-financed studies have at least one author with stocks in the funding company and it is possible that this direct financial conflict and incentive might affect judgment and hamper scientific integrity of the data, with strategic and moral reason to inflate advice [15, 16]. Bias related to COI might be related to unconscious motivational processes [17, 18]. Several mechanisms on how positive outcomes can be achieved have been proposed, for example by statistical analysis such as randomization procedures, parametric analyses, run-in enrichments technique, and dichotomizing of ordered data [19]. This study highlights the strong connection between industry and COI and it can be discussed if this tight bond might jeopardize scientific validity of the studies. Studies report COI balance between science, advertisement, and revenue.

Translating our findings into a policy change, we do believe it important that the community is aware of the actual disclosure rates in the studies and type of financial disclosures. Since different COI domains might be related to favorable outcomes, this is a useful tool to critically appraise trial reports. Authorization of stock ownership by trial authors might still be accepted but needs to be discussed in a broader context in the future.

Limitations of this study needs to be addressed. As we only considered self-declared COI, there is a potential of underreporting of the actual COI that might exist as shown previously [20].

Furthermore, we included all randomized studies that mentioned the term “random” in the methods section. Since this can include studies with vague description of their randomization procedure, this is a further limitation of our study [21]. Study selection always introduces bias and our selection of trials published in highly cited journals with a high proportion of industry-sponsored trials might have introduced bias. It is possible that for-profit financed RCTs published in high impact journals are the first major trials published for a novel treatment and early positive trials tend to have larger effect sizes than later ones [22]. Thus, the generalizability of COI to lower impact journals cannot be ascertained. Further, we included only parallel-group RCTs with binary outcomes such as survival, progression-free survival and myocardial infarction, excluding studies with continuous variables since we consider that it is relatively more difficult to obtain a statistical significant result for binary outcomes since they are more often related to a clinical endpoint rather than surrogate endpoint [23] and to establish more comparable study groups. Furthermore, parallel-group RCTs were exclusively included to simplify the assessment of a favorable outcome, compared with a multi-arm RCT. However, we acknowledge that this further limits the generalizability of the study.

The prevalence of royalties and patents among authors was low in our cohort and not associated with positive outcome.

## Conclusions

COI in for-profit funded RCTs is extensive with several COI domains associated with favorable outcomes: because the factors related to COI are not fully independent, a multivariable analysis should be cautiously interpreted. However, only stock ownership from the funding company among authors was related to a favorable outcome. Future studies should address the structure and mechanism on how stock ownership among authors in RCTs affect outcome.

## Additional files

Additional file 1: PRISMA checklist. (DOC 62 kb)

Additional file 2: Review protocol and raw data. (XLS 194 kb)

## Abbreviations

COI: Conflict of interest
RCT: Randomized controlled trial
